# On Some Points in the Operation for Hare-lip

**Published:** 1871-02

**Authors:** F. C. Hotz


ARTICLE V.—*On some Points in the Operation for Hare-lip.* A Paper read before the Chicago Medical Society, by F. C. HOTZ, M.D.

The operation for hare-lip is very old, and has passed through many changes and discussions. So the surgeons, at one time, had a lively debate on the question whether the edges of a hare-lip should be pared with knives or scissors. At some other time, again, the good result of the operation was chiefly attributed to the pins used, and it was considered as a matter of the greatest importance whether these pins were of iron, steel, silver, gold, or platina, and whether the point was flat, rounded, or triangular—and you know well enough what an immense number of different hare-lip pins has been devised. Thus wasting much time and talent, in paying his whole attention to very unessential things, the surgeon neglected the most important point of the operation. And the time is not so very far back since we have been convinced that the success of a plastic operation does not depend on the material of the sutures, but that the direct adhesion is best secured by thoroughly excising and accurately uniting the edges.

The age at which the operation should be done, has also been the topic of discussion for some time. Now, however, surgeons generally, I think, agree that in strong, healthy children, the operation should be performed in the first three months; at least, before dentition, because an early closure of the fissure in the lip has proved to best remedy that flattening of the nose so commonly observed with hare-lips.

Thus, taking the question about the time best appropriate for the operation to be settled, there are three other points of interest in regard to the final result of the operation.

1. The way of paring the edges of the fissure.
2. The kind of stitches.
3. The treatment after the operation.

On each of these points there is still more or less difference of opinion among surgeons, as they claim to get equally good results by different ways of operating. I do not deny this fact, to be sure; but when a man wishes to get to any point, he does not ask only by which way he can get there, but he inquires for the shortest way. And so we must not be satisfied with a good result, but we must ask, which kind of operation leads to a full success, by the simplest and safest way. And from this standpoint, gentlemen, I wish you would receive the following remarks on the features above mentioned.

### I. PARING THE EDGES.

As I have already stated, this must always be done freely and carefully, in order to give any prospect for direct adhesion. But in regard to the permanent form of the lip, various ways have been tried to obviate all deformity, especially that very unpleasant, prolabial notch, so frequently left after an operation. You all are aware that this notch is occasioned by the cicatrice shrinking principally in its longitudinal direction. And you know also, that, as a rule, it is left if the incisions have been made entirely straight. But it is very likely to occur, also, in a somewhat broad cleft, although the incisions have been concave. *Malgaigne*, therefore, tried a more effective way to compensate the shortening of the cicatrice by cutting, at the lower end of the fissure, two small, angular flaps, which, when united, at first formed a small protuberance, that afterwards, as he thought, would shrink down just to a level with the prolabium. It is, however, impossible to know in advance exactly how much a cicatrice will shrink, and consequently we can never be sure whether we get just the accurate size of these flaps. They may be either too small, when the lip will become notched, or too large, when they will leave a protuberance as unseemly as a notch.

Now we have not to struggle with any of these uncertainties by following a plan of excising the edges of hare-lips, first suggested by *Mirault* and *Langenbeck* (French and German surgeons), a method which seems to me less known and practiced in this country than it really deserves to be.

The original directions were, that one small, angular flap (like those of Malgaigne's) was formed at the lower end of the better developed side of the fissure; the other edge was then pared off straight down in its entire length, and finally the prolabium of this side was refreshed so far as to correspond to the shape and size of that angular flap. But as such a small flap, sloping down to a thin edge, has not the best chances of healing on by first union, it is very advisable to cut off its thinnest part in a right angle, and excise the prolabium of the opposite side also in a right angle, in conformity with this shorter rectangular flap.

This method perhaps requires a little more care and time than the other ones, but with some skill and practice it can be quickly executed in children as well as adults. It affords the same, if not a greater, safety of healing by first union, and it is superior to all other methods by completely preventing the prolabial notch; which it accomplishes, not by lengthening the cicatrice in order to compensate its shrinking (as Malgaigne and others did,) but by separating the wound in the lip itself from that in the prolabium. The consequence of which separation is, that the greater labial cicatrice—should it shrink ever so much—cannot affect the prolabium, while the scar in the latter is too minute to show any visible contraction. In this manner, as you see, the success of the operation is rendered entirely independent of all changes which subsequently may take place in the cicatricious tissue; and for this reason I consider *Mirault's* and *Langenbeck's* method preferable to any other.

2. UNITING THE PARED EDGES.

For a long time the twisted suture was the only kind used to unite the edges of a hare-lip; and it is predominant still, and considered by many surgeons (Dr. Gross e. g.) the only kind that should be used. Some surgeons, however, in Germany (Langenbeck, Simon, and others) as well as in England (Erichsen), have, in the last years, used the simple sutures with very good success. So says Erichsen in his "Science and art of Surgery" (p. 69):

"During the last few years I have been in the habit of treating hare-lips of all kinds, double as well as single, with the simple interrupted suture alone, without using any pins. I have in this way treated most successfully many cases in children, whose ages have varied from a few days to four years, with most satisfactory results, and with less marking of the lip than I have ever seen attend union by means of the twisted suture, to which I now generally prefer it, as being *equally safe*, *more simple*, and followed by less scarring of the lip."

I fully agree with these remarks. During five years of practice in the University Hospital at Heidelberg (Germany), I have had sufficient opportunity of comparing the value of both kinds of suture; for Chelius always applied the pins, while his successor,

Prof. Simon, used the simple sutures alone. And from these observations I have been convinced that the children suffered more from the pins, since they prevented them from sucking, and caused considerable pain, and were more likely to set up an excessive inflammation, that might destroy the fresh adhesion again by suppuration. Therefore the surgeon must watch their effect most carefully, and has to take out the pins forty-eight hours after the operation, at latest. This proceeding, even by the utmost care, cannot be done without the child screaming and crying—precisely what we have to prevent, by all means. Now, on the other hand, the simple sutures (of any fine material whatever) may be left in for four to eight days without doing any harm; they don't hinder the child from taking the breast, nor do they provoke any injurious inflammation; therefore they need not be removed until the cicatrice will be firm enough to resist all dragging upon during screaming. Why, then, if the simple suture has all these advantages, was the more complicated twisted suture ever used? Because it was thought the only effectual means by which all injurious tension could be obviated, and the raw edges kept in accurate apposition. Now, no surgeon ever thought of using pins in treating a cleft palate, where the conditions are pretty much the same. And the facts prove that we can succeed with the simple sutures, especially if we apply them in a double row, according to their double purpose to relax and to unite the edges. Thus, near the lower end of the fissure, and wherever a great tension is to be obviated, we introduce a suture which incloses in its loop the entire thickness of the lip, 1½ lines from each edge. If we tie these sutures, they will surely release the wound from any tension; but the mucous lining and the skin would very likely double up. Therefore, to adjust the raw edges most accurately, some other sutures must be introduced in the space between the former, in such a way that the needle (strongly curved) is passed in close at the border of the wound, and carried through the lip in a short semi-circle, and brought out again without transfixing the mucous lining.

The simple sutures introduced in the manner just described, will unite the edges of any kind of hare-lips as safely as the pins, and all tension which cannot be removed by them without danger of their cutting through too soon, will better be obviated by lateral incisions, or by detaching the lips from the gums.

### 3. DRESSING AFTER THE OPERATION.

Dieffenbach has already said (Operative Chirurgie): "Any dressing after this operation is not practical; instead of securing the success, it often frustrates it; many lips have been lost by the undue pressure of strips of adhesive plaster." And I think he was right. Neither plaster-strips, nor collodion, nor cheek compressors, are any reliable means to obviate dragging upon the wound. Nothing is required but to keep the parts cool and the child as quiet as possible.

And so I have come to the end of my paper, whose object was to point out the safest, simplest, and least troublesome way of treating hare-lips. The result of which investigation I may sum up in these words:

The edges shall be pared according to Mirault and Langenbeck's method, and brought together with a double row of interrupted sutures. The child is nursed in the same manner as before the operation, and everything avoided that could make it uneasy. Therefore the wound is not cleaned nor examined by the surgeon until after three to five days, or later; the adhesion will be sufficiently firm. Then the sutures may be taken out; yet no applications of any kind are made, but the child let alone with his mother. Because we had better remove all causes for screaming and crying, than to try to obviate its effect, *i. e.*, the dragging upon the wound, by means which will always prove a failure.

